# Impaired Gonadotropin-Lowering Effects of Metformin in Postmenopausal Women with Autoimmune Thyroiditis: A Pilot Study

**DOI:** 10.3390/ph16070922

**Published:** 2023-06-24

**Authors:** Robert Krysiak, Marcin Basiak, Grzegorz Machnik, Bogusław Okopień

**Affiliations:** Department of Internal Medicine and Clinical Pharmacology, Medical University of Silesia, 40-752 Katowice, Poland; mbasiak@sum.edu.pl (M.B.); gmachnik@sum.edu.pl (G.M.); mbkdokop@mp.pl (B.O.)

**Keywords:** autoimmunity, FSH, insulin resistance, LH, menopause, metformin, pituitary, thyroid

## Abstract

Metformin has been found to reduce elevated gonadotropin levels. Hashimoto’s thyroiditis is the most common thyroid disorder in iodine-sufficient areas, and it often develops in postmenopausal women. The aim of this study was to investigate whether autoimmune thyroiditis determines the impact of metformin on gonadotrope secretory function. Two matched groups of postmenopausal women were studied: 35 with euthyroid Hashimoto’s thyroiditis (group A) and 35 without thyroid disorders (group B). Throughout the study, all participants received oral metformin (2.55–3 g daily). Plasma glucose, insulin, gonadotropins, estradiol, progesterone, thyrotropin, free thyroid hormones, prolactin, adrenocorticotropic hormone, insulin-like growth factor-1, hsCRP, thyroid peroxidase, and thyroglobulin antibody titers were measured at the beginning of the study and six months later. At entry, both groups differed in thyroid peroxidase antibody titers, thyroglobulin antibody titers, and hsCRP levels. In group A, baseline antibody titers correlated positively with hsCRP and negatively with insulin sensitivity. Although metformin improved glucose homeostasis and reduced hsCRP levels in both study groups, these effects were more pronounced in group B than in group A. Only in group B did metformin decrease FSH levels and tend to reduce LH levels. Thyroid antibody titers and the levels of the remaining hormones did not change throughout the study. The impact of metformin on gonadotropin levels correlated with their baseline values and the degree of improvement in insulin sensitivity, as well as with the baseline and treatment-induced reduction in hsCRP. Moreover, the impact on gonadotropins and insulin sensitivity in group A depended on baseline antibody titers. The obtained results indicate that coexisting autoimmune thyroiditis impairs the gonadotropin-lowering effects of metformin in postmenopausal women.

## 1. Introduction

Metformin, the first-choice drug for type 2 diabetes and other insulin-resistant states [1], was found to affect the secretory function of overactive thyrotropes [2,3], lactotropes [4,5], and gonadotropes [6,7,8,9,10]. The drug decreased FSH levels in postmenopausal women [6,7], LH levels in women with polycystic ovary syndrome [8,9], and the levels of both gonadotropins in men with hypergonadotropic hypogonadism [10]. The pituitary effects seem to result from the accumulation of significant amounts of metformin in the pituitary [11], probably owing to the lack of the blood–brain barrier in this brain area [12]. At least in rodent pituitary cells, the inhibitory effect of metformin on gonadotropin secretion is mediated by the stimulation of 5′-adenosine-monophosphate-activated protein kinase (AMPK) [13].

Autoimmune thyroiditis, also referred to as Hashimoto’s thyroiditis, is the most frequent cause of hypothyroidism in developed countries, the most prevalent organ-specific autoimmune disorder, and one of the most common human disorders [14,15]. The autoimmune thyroid disorder is much more common in women than in men (a female-to-male ratio of 5–10:1), with the prevalence rising with age and being the highest in individuals older than 60 years [15,16]. Autoimmune thyroid disease, even in euthyroid, non-obese patients, is associated with a higher prevalence of insulin resistance, metabolic syndrome, and other disturbances of glucose homeostasis [17]. The risk of developing insulin resistance, type 2 diabetes, and prediabetes is also increased after menopause [18]. This means that many postmenopausal women are characterized by the concomitant presence of Hashimoto’s thyroiditis and insulin-resistant states, as well as that many postmenopausal women with thyroid autoimmunity require treatment with metformin.

To the best of our knowledge, only one study has assessed the role of autoimmune thyroiditis in the pituitary effects of metformin. The decrease in thyrotropin levels was observed in subjects with hypothyroidism of both autoimmune and non-autoimmune origin and did not correlate with thyroid antibody titers [2]. The paucity of data encouraged us to investigate whether the pituitary effects of metformin differ between postmenopausal women with thyroid autoimmunity and without thyroid disorders.

## 2. Results

One woman (assigned to group A) dropped out because of the adverse effects associated with metformin treatment (vomiting and diarrhea). Another woman (assigned to group B) was withdrawn because of non-compliance. No adverse effects were reported in the remaining 68 women (34 in each group) who completed the study and were subjected to statistical analyses. All analyzed patients complied with the treatment recommendations and adhered to the recommendations on diet and physical activity. A post hoc power calculation showed that the study had sufficient statistical power (0.90). The daily metformin dose was similar in groups A and B (2.80 ± 0.22 g vs. 2.78 ± 0.23 g; *p* = 0.7152).

At study entry, both groups differed in TPOAb titers, TgAb titers, and hsCRP levels. There were no differences between both groups in age, smoking, body mass index, blood pressure, glucose homeostasis markers (glucose, insulin, and HOMA1-IR), thyrotropin, free thyroid hormones, FSH, LH, estradiol, progesterone, anti-Müllerian hormone, prolactin, ACTH, or insulin-like growth factor-1 (Table 1 and Table 2).

Although metformin decreased glucose, insulin, and hsCRP levels, and reduced HOMA1-IR in both study groups, all these effects were more pronounced in group B than in group A. In group B, metformin decreased FSH levels and tended (*p* = 0.0523) to reduce LH levels. In group A, there were no differences between the follow-up and baseline gonadotropin levels. The thyroid antibody titers and circulating levels of the remaining hormones did not change throughout the study (Table 2 and Table 3). At the end of the study, both groups differed in glucose, insulin, HOMA1-IR, gonadotropins, and hsCRP (Table 2). Both study groups differed in percentage changes from baseline in glucose, insulin, HOMA1-IR, gonadotropins, and hsCRP (Table 3).

In the women with prediabetes refusing metformin treatment but complying with the lifestyle intervention program, there were no differences between the baseline and follow-up values of glucose homeostasis markers, antibody titers, hormone levels, and hsCRP levels (Table 4 and Table 5).

At entry, the thyroid antibody titers in group A positively correlated with hsCRP levels (TPOAb: r = 0.51, *p* < 0.0001; TgAb: r = 0.47, *p* = 0.0001), insulin levels (TPOAb: r = 0.37, *p* < 0.0058; TgAb: r = 0.31, *p* = 0.0402), and HOMA1-IR (TPOAb: r = 0.40, *p* = 0.0003; TgAb: r = 0.38, *p* = 0.0041). The impact of treatment on gonadotropin levels positively correlated with baseline gonadotropin levels (group A—FSH: r = 0.35, *p* = 0.0125; LH: r = 0.28, *p* = 0.0402; group B—FSH: r = 0.48, *p* = 0.0001; LH: r = 0.40, *p* = 0.0012) and with the treatment-induced decrease in hsCRP (group A—FSH: r = 0.41, *p* = 0.0004; LH: r = 0.37, *p* = 0.0085; group B—FSH: r = 0.50, *p* < 0.0001; LH: r = 0.42, *p* = 0.0004), as well as negatively correlating with baseline hsCRP levels (group A—FSH: r = −0.42, *p* = 0.0006; LH: r = −0.32, *p* = 0.0230; group B—FSH: r = −0.46, *p* = 0.0002; LH: r = −0.35, *p* = 0.0115). The impact on FSH levels positively correlated with the treatment-induced reduction in insulin (group A: r = 0.37, *p* = 0.0087; group B: r = 0.39, *p* = 0.0022) and in HOMA1-IR (group A: r = 0.40, *p* = 0.0004; group B: r = 0.43, *p* = 0.0004). In group A, but not in group B, the effect on gonadotropins, insulin, and HOMA1-IR inversely correlated with the baseline titers of TPOAb (FSH: r = −0.42, *p* = 0.0004; LH: r = −0.32, *p* = 0.0288; insulin: r = −0.37, *p* = 0.0086; HOMA1-IR: r = −0.34, *p* = 0.0134) and TgAb (FSH: r = −0.38, *p* = 0.0049; LH: r = −0.28, *p* = 0.0462; insulin: r = −0.29, *p* = 0.0411; HOMA1-IR: r = −0.31, *p* = 0.0256). Moreover, the impact of treatment on hsCRP inversely correlated with baseline hsCRP levels (group A: r = −0.39, *p* = 0.0016; group B: r = −0.34, *p* = 0.0165), and in group A, it inversely correlated with baseline antibody titers (TPOAb: r = −0.37, *p* = 0.0080; TgAb: r = −0.32, *p* = 0.0268). All other correlations were not significant.

## 3. Discussion

In line with previous studies [6,7], metformin administered to postmenopausal women without thyroid disorders decreased circulating gonadotropin levels, and this effect was stronger for FSH than for LH. This relative difference in the impact on both gonadotropins probably reflects the baseline differences in gonadotropin production. In line with this explanation, the treatment-induced changes in FSH and LH levels correlated with their baseline values. Moreover, the drug did not affect the plasma levels of the remaining anterior pituitary hormones (thyrotropin, prolactin, and ACTH) and insulin-like growth factor-1, mediating most biological effects of the growth factor [19], all of which were within the reference range. This finding seems to be clinically important. Animal studies showed that FSH-blocking antibodies increased bone mass, reduced body fat, enhanced thermogenesis, and prevented neurodegeneration [20,21]. Thus, the FSH-lowering effect of metformin in women after menopause may attenuate bone loss, obesity, and early cognitive decline. A beneficial effect on cognition may be additionally potentiated by a small decrease in LH, which was found to induce neurodegenerative changes, such as those seen in Alzheimer’s disease [22]. The decrease in gonadotropin levels cannot be attributed to the impact of the dietary intervention and/or physical activity because both FSH and LH concentrations remained at similar levels in the metformin-naïve women complying with the lifestyle intervention program and observed during the same period of time as the women receiving metformin therapy.

Based on these results, three conclusions may be drawn. Firstly, beyond the metabolic effects, metformin was found to stimulate osteoblastogenesis and to decrease bone resorption [23], as well as to alleviate age-related cognitive dysfunction [24]. These effects may be in part associated with the impact on overactive gonadotropes. Secondly, because the follow-up gonadotropin levels were still higher than those in women of reproductive age, the impact on bone mass and cognitive function mediated by gonadotrope secretory function is probably moderate. However, it should be taken into consideration when choosing a treatment strategy in insulin-resistant postmenopausal women at high risk of osteoporosis or dementia. Lastly, the only moderate effect and correlations with baseline gonadotropin levels suggest that metformin treatment does not lead to gonadotrope hypofunction, the clinical significance of which after menopause has not been determined.

However, the most important finding of the present study is that the gonadotropin-lowering effect of metformin was absent in the case of coexisting autoimmune thyroiditis. The current study exclusively included patients with thyrotropin and free thyroid hormone levels within the reference range. Moreover, to eliminate the mild thyroid dysfunction resulting from thyroid lymphocytic infiltration, both groups were matched for thyrotropin and free thyroid hormones (as well as for gonadotropins and glucose homeostasis markers). Lastly, owing to the strict inclusion and exclusion criteria, the between-group differences in metformin action cannot be explained by comorbidities and concurrent therapies. Therefore, our findings seem to reflect the presence of thyroid autoimmunity itself.

The obtained results do not provide a clear explanation of the mechanisms by which autoimmune thyroiditis impairs the gonadotropin-lowering effects of metformin. Our findings suggest that differences in the action on plasma gonadotropins between both study groups may be associated with counterbalancing the effect of metformin treatment by the mechanisms underlying systemic inflammation. Hashimoto’s thyroiditis is characterized by elevated circulating levels of hsCRP, proinflammatory cytokines, and other indices of systemic inflammation [25]. This well explains why hsCRP levels were higher in women with autoimmune thyroiditis than in women without this disorder. However, both groups also differed in the impact of metformin on systemic inflammation. Unlike women without thyroid pathology in whom the drug reduced hsCRP, the metformin treatment of subjects with autoimmune thyroiditis did not affect the plasma levels of this protein, reflecting a neutral effect on antibody titers, observed in the current study and reported previously by our research team [26,27]. This finding is in contrast with the results of recent studies, mainly preclinical ones, which showed that metformin exerted pluripotential anti-inflammatory properties in cardiovascular diseases, diabetes, other metabolic disorders, kidney disorders, neurodegenerative diseases, and tumors [28,29]. The most probable explanation for this discrepancy is that metformin is able to exert its anti-inflammatory effect only in states associated with mild systemic inflammation and not in more advanced stages of system inflammation, characterizing women with autoimmune thyroid disease [25]. In line with this explanation, the impact of metformin on hsCRP inversely correlated with baseline hsCRP levels and antibody titers. Although we cannot rule out that our study might have been underpowered to detect small changes in hsCRP levels (the impact on hsCRP was not the primary endpoint), systemic inflammation associated with Hashimoto’s thyroiditis persisted despite metformin treatment, and it might have prevented the gonadotropin-lowering effects of this agent.

Some findings suggest that metformin and mediators of thyroid autoimmunity may interact at the level of pituitary AMPK. Gonadotropin-secreting cells are characterized by a greater expression of AMPK than other types of anterior pituitary cells [13]. Pituitary AMPK was found to mediate the impact of metformin on basal gonadotropin secretion, and on gonadotropin secretion induced by gonadotropin-releasing hormone and activin [13]. AMPK activation is inversely associated with inflammation, and it correlates with its degree [30]. Lastly, various proinflammatory cytokines were found to suppress AMPK activation [31]. Because the major sources of AMPK in circulation are the liver, muscle cells, and adipose tissue, and its production depends significantly on cellular energy homeostasis [32], a random assessment of AMPK in plasma or serum poorly reflects AMPK activity in the pituitary gland. We also cannot exclude that the decrease in gonadotropin production is partially indirect and results from the metformin action on gonadotropin-releasing hormone production in the arcuate nucleus and/or its release in the median evidence, the hypothalamic structures with abundant expressions of AMPK [33]. However, gonadotropin-releasing hormone is secreted into the portal system in a pulsatile manner [34], and, therefore, the circulating levels of this hormone do not provide information on its production and release. Thus, future experimental and cellular studies are needed to establish a definitive link between the obtained results and the impact of metformin treatment on AMPK.

Although metformin was found to reduce progesterone and estradiol production by rat granulosa cells [35], the impact of this drug on ovarian function in postmenopausal women was neutral. Irrespective of study group, the drug did not affect the circulating levels of estradiol, progesterone, and anti-Müllerian hormone, a widely used marker of functional ovarian reserve [36]. Very low estradiol levels throughout the study are also an argument against increased peripheral aromatization of ovarian and adrenal androgens in the adipose tissue, muscles, liver, skin, and breast tissues, despite the stimulatory effect of metformin on aromatase activity [37] and the important role of extra-gonadal production as a source of estrogens in postmenopausal women [38]. These discrepancies may be explained by age-related changes in ovarian activity and high concentrations of metformin in in vitro studies exceeding the plasma levels reached after treatment with therapeutic doses of this agent [39].

Another interesting finding of the present study is the between-group differences in metformin action on insulin sensitivity and their association with thyroid antibody titers. Although the drug reduced insulin levels and HOMA1-IR, an established marker of insulin sensitivity [40], in both study groups, these effects were more pronounced in individuals without thyroid disorders than in subjects with autoimmune thyroiditis. Because metformin administered to subjects at high risk for type 2 diabetes prevented or delayed the development of diabetes [41], the obtained results suggest that the risk of progression to diabetes may be greater in individuals with coexistent Hashimoto’s thyroiditis. Interestingly, the decrease in insulin and HOMA1-IR correlated with the FSH-lowering effect of metformin, indicating that these effects are interrelated. This finding is in line with suggestions that the reduction in increased FSH levels may be metabolically beneficial [20]. The unfavorable effect of thyroiditis on the metabolic properties of metformin may be associated with the impact of proinflammatory cytokines and systemic inflammation on the GLUT4 transporter, the major facilitative glucose transporter in skeletal muscles, adipose tissue, and other peripheral tissues [42]. In line with this explanation, hsCRP levels correlated with both thyroid antibody titers and with insulin levels and HOMA1-IR. Moreover, GLUT4 translocation and membrane expression were blocked by interleukin-1ß, tumor necrosis factor-α, and interferon-γ [43,44,45]. An increased production of all these cytokines by proinflammatory cells is observed in euthyroid women with autoimmune thyroiditis [46].

In light of previous findings, the obtained results allow us to draw some practical conclusions. Firstly, euthyroid Hashimoto’s thyroiditis, despite normal levels of thyrotropin and free thyroid hormones, seems to be a disorder with important clinical implications. An increasing number of studies suggest an association between autoimmune thyroiditis and various aspects of cognitive dysfunction, such as difficulties with memory, focusing attention, and the slowing down of thinking processes, observed even in subjects with normal thyrotropin and thyroid hormone levels [47,48]. Hashimoto’s thyroiditis also seems to affect bone metabolism, leading through both endocrine and immune pathways to a decrease in bone mass and strength [49]. Moreover, euthyroid autoimmune thyroid disease is associated with impaired insulin sensitivity [50]. Although no study has compared various age groups of subjects with autoimmune thyroid disease, postmenopausal women with Hashimoto’s thyroiditis may be particularly prone to the development of these complications due to the additive effects of postmenopause and thyroid autoimmunity. Secondly, providing that the decrease in elevated gonadotropin production is desirable, euthyroid women with Hashimoto’s thyroiditis may benefit to a lesser degree from metformin treatment than the remaining populations of postmenopausal women. The risk of cognitive impairments, bone loss, and metabolic complications in this population despite treatment may be greater than that in their peers without thyroid pathology. Thirdly, inverse correlations between the impact of metformin on gonadotropin levels and baseline HOMA1-IR, the association between hypothyroidism and insulin resistance [17], and the increased risk of dementia in subjects with hypothyroidism [51] indicate that the unfavorable effect of autoimmune thyroid disease on the pituitary and metabolic effects of metformin may be greater if Hashimoto’s thyroiditis results in thyroid hypofunction. Lastly, the correlations between thyroid antibody titers and hsCRP, between hsCRP and the impact on gonadotropin levels, between antibody titers and insulin sensitivity, and between antibody titers and the changes in insulin and HOMA1-IR suggest that the effect of metformin on gonadotrope function and glucose homeostasis in individuals with thyroid autoimmunity may be potentiated by concomitant treatment with levothyroxine or with other agents decreasing thyroid antibody titers and having systemic anti-inflammatory properties (exogenous vitamin D, selenomethione, and/or myo-inositol) [52,53]. However, the putative implications of autoimmune thyroiditis and metformin treatment for bone health, cognitive function, obesity, and metabolism require further investigation.

Some study shortcomings have to be pointed out. Because our single-center study included a relatively small number of patients, the obtained results should be interpreted with caution. A larger sample size would increase the statistical power and generalizability of our findings. The study was non-randomized and did not include a group receiving a placebo. The population inhabiting the Upper Silesia (the area where the study was carried out) is characterized by low selenium status [54] and an adequate iodine intake [55]. Hence, it remains unsolved whether the impact of metformin is similar in individuals in whom selenium supply is sufficient and/or iodine supply is insufficient. Although the inclusion of only postmenopausal women enabled us to obtain a relatively homogenous study population, the lack of men and women with elevated FSH levels of other origin does not allow us to conclude about the impact of autoimmune thyroiditis on metformin action in these groups. The groups were matched only for age, insulin sensitivity, and hormone levels, while other factors such as family circumstances, comorbidities, and the short-term use of other drugs were not adequately accounted for. Therefore, these factors could have potentially influenced the outcomes and limited the interpretation of the results. Finally, although the research design limited the impact of random diurnal, seasonal, and analytical variations in the measured variables, we cannot totally exclude the regression-toward-the-mean effect.

## 4. Materials and Methods

Written informed consent was obtained from all patients, and the study protocol was approved by the institutional committee on human research, ensuring that it conformed to the ethical guidelines of the Declaration of Helsinki.

### 4.1. Study Population

The study population was recruited among postmenopausal women (50–70 years) with prediabetes. All women met the following criteria of age-related (natural) postmenopause: (1) the absence of menstrual periods for at least 12 months, (2) plasma FSH levels above 30 IU/L, and (3) a plasma estradiol concentration below 30 pg/mL. Prediabetes was defined as fasting glucose between 5.6 and 7.0 mmol/L (100–125 mg/dL) and/or 2 h postchallenge glucose between 7.8 and 11.0 mmol/L (140–199 mg/dL). Only women complying with the lifestyle modification for at least 12 weeks were included. The study population consisted of two groups of women. Group A included 35 women with euthyroid autoimmune thyroiditis, defined as plasma titers of thyroid peroxidase antibodies (TPOAb) above 100 U/mL, the typical sonographic appearance of autoimmune thyroiditis, and circulating levels of thyrotropin and free thyroid hormones within the reference range (thyrotropin between 0.4 and 4.5 mU/L, free thyroxine levels between 10.2 and 21.5 pmol/L, and free triiodothyronine between 2.3 and 6.4 pmol/L). In turn, group B (*n* = 35) was selected from a larger group of postmenopausal women (*n* = 82) with no evidence of thyroid disease. The aim of this selection was to obtain two study groups matched for age; insulin sensitivity; and circulating levels of gonadotropins, thyrotropin, and free thyroid hormones. A preliminary sample size calculation showed that at least 29 patients in each treatment group were needed to detect a 20% difference in the primary endpoint (plasma gonadotropin levels) with the power of 80% and a significance level of 5%. To limit the impact of possible seasonal variations in the investigated variables and seasonal confounds, participants were recruited either between January and February (*n* = 36) or between June and July (*n* = 34).

The subjects were excluded if they met at least one of the following criteria: other endocrine and/or autoimmune disorders, chronic inflammatory disorders, kidney insufficiency, impaired liver function, malabsorption syndromes, other serious disorders, and poor patient compliance. We also excluded individuals treated within the last six months with any prescription or over-the-counter drug for more than a week.

### 4.2. Study Design

Over the entire study period (six months), all patients received metformin. The dose of this drug was gradually up-titrated (every 5–7 days, depending on tolerance) from a starting dose of 850 mg once daily, to 850 mg twice daily, and to the final dose (850–1000 mg three times a day), which was administered for the remaining period of time. The participants were also requested to comply with the goals of lifestyle modification (total fat intake <30% of total energy intake, saturated fat intake <7% of energy consumed, cholesterol intake <200 mg per day, fiber intake ≥15 g per 1000 kcal, and ≥150 min of moderate-intensity aerobic physical activity per week). Treatment compliance was assessed every six weeks by counting the number of tablets returned. Compliance with non-pharmacological recommendations was assessed via an analysis of individual dietary questionnaires and of diaries in which the participants continuously recorded all their activities.

### 4.3. Metformin-Naïve Patients

To investigate whether the non-pharmacological intervention affects the obtained results, we performed a parallel study comparing the baseline and follow-up values of glucose metabolism markers, antibody titers, hormones, and high-sensitivity C-reactive protein (hsCRP) in prediabetic women with autoimmune thyroiditis (*n* = 29) and without thyroid pathology (*n* = 29), meeting all inclusion criteria but refusing metformin therapy. These women complied with the same lifestyle intervention for six months (and for at least 12 weeks before the beginning of the study) as the participants of the main study but did not receive pharmacotherapy.

### 4.4. Laboratory Assays

Venous blood samples were drawn from the antecubital vein in the morning in the seated position after the overnight fasting (at least 12 h after the last meal) at study entry and six months later. All laboratory assays were carried out in duplicate. Plasma glucose was assessed using a COBAS Integra 400 Plus analyzer (Roche Diagnostics, Basel, Switzerland). Circulating concentrations of insulin, FSH, LH, thyrotropin, prolactin, estradiol, progesterone, and anti-Müllerian hormone (only in samples of 17 patients in each group), as well as the titers of TPOAb and thyroglobulin antibodies (TgAb), were measured using acridinium ester technology (ADVIA Centaur XP Immunoassay System, Siemens Healthcare Diagnostics, Munich, Germany). Plasma levels of adrenocorticotropic hormone (ACTH), insulin-like growth factor-1, and hsCRP were measured using solid-phase enzyme-labeled chemiluminescent immunometric assays (Immulite, Siemens, Munich, Germany). The homeostasis model assessment 1 of insulin resistance index (HOMA1-IR) was calculated on the basis of circulating glucose and insulin levels using the following equation: plasma glucose (mmol/L) × plasma insulin (mIU/L)/22.5.

### 4.5. Statistical Analysis

All data were logarithmically transformed in order to obtain an approximately normal distribution. Student’s unpaired *t* tests were used to compare both groups, while Student’s paired *t* tests were applied to compare the differences between the means of variables within the same study group. The χ^2^ test was employed to compare the proportional data. Correlations between the investigated variables were assessed using Pearson’s r tests for two continuous variables; the Phi coefficient for one continuous and one categorical variable; and point-biserial for two categorical variables. Differences were regarded as statistically significant if the *p*-values corrected for multiple testing were below 0.05.

## 5. Conclusions

In conclusion, metformin administered to insulin-resistant postmenopausal women reduced FSH levels and tended to reduce LH levels, and this effect correlated with baseline gonadotropin levels. The gonadotropin-lowering properties of metformin were absent in postmenopausal women with euthyroid Hashimoto’s thyroiditis. This finding, as well as the relatively weak effect of metformin on glucose homeostasis in the case of coexistent autoimmune thyroid disease, suggests that postmenopausal women with Hashimoto’s thyroiditis are worse candidates for metformin treatment than other populations of insulin-resistant postmenopausal women. Due to the numerous limitations resulting from the pilot nature of this study and the lack of a clear mechanistic explanation, further research is needed to confirm our findings in a larger and more diverse population and to understand their clinical implications.

## Figures and Tables

**Table 1:** Baseline characteristics of patients.

Variable	Group A	Group B	*p*-Value
Number (*n*)	34	34	-
Age (years)	59 ± 5	60 ± 6	0.4580
Smokers (%)	38	35	-
Body mass index (kg/m^2^)	24.4 ± 4.9	23.7 ± 5.1	0.5658
Systolic blood pressure (mmHg)	134 ± 12	132 ± 14	0.5293
Systolic blood pressure (mmHg)	85 ± 4	84 ± 5	0.3658

Group A: postmenopausal women with autoimmune thyroiditis. Group B: postmenopausal women without thyroid pathology. Unless otherwise stated, the data are presented as the mean ± standard deviation.

**Table 2:** The effect of metformin on glucose homeostasis markers, thyroid antibodies, hormones, and low-grade inflammation in the study population.

Variable	Group A	Group B	*p*-Value (A vs. B)
Glucose (mmol/L)			
*Baseline*	6.00 ± 0.69	6.06± 0.65	0.7134
*Follow-up*	5.67± 0.61	5.38 ± 0.50	**0.0357**
*p-value (follow-up* vs. *baseline)*	**0.0406**	**<0.0001**	-
Insulin (mU/L)			
*Baseline*	15.0 ± 4.0	14.6 ± 3.8	0.6738
*Follow-up*	11.8 ± 3.0	8.5 ± 2.5	**<0.0001**
*p-value (follow-up* vs. *baseline)*	**0.0004**	**<0.0001**	-
HOMA1-IR			
*Baseline*	4.0 ± 1.1	3.9 ± 1.0	0.6962
*Follow-up*	3.0 ± 0.7	2.0 ± 0.6	**<0.0001**
*p-value (follow-up* vs. *baseline)*	**<0.0001**	**<0.0001**	-
TPOAb (IU/mL)			
*Baseline*	912 ± 291	15 ± 11	**<0.0001**
*Follow-up*	810 ± 210	14 ± 12	**<0.0001**
*p-value (follow-up* vs. *baseline)*	0.1022	0.7213	-
TgAb (IU/mL)			
*Baseline*	860 ± 302	18 ± 10	**<0.0001**
*Follow-up*	750 ± 248	16 ± 15	**<0.0001**
*p-value (follow-up* vs. *baseline)*	0.1055	0.5188	-
Thyrotropin (mIU/L)			
*Baseline*	2.4 ± 1.0	2.2 ± 0.9	0.3892
*Follow-up*	2.3 ± 1.0	2.0 ± 0.9	0.1980
*p-value (follow-up* vs. *baseline)*	0.6814	0.3628	-
Free thyroxine (pmol/L)			
*Baseline*	14.4 ± 2.2	15.0 ± 2.5	0.2973
*Follow-up*	14.8 ± 2.4	15.3 ± 2.8	0.4320
*p-value (follow-up* vs. *baseline)*	0.4763	0.6427	-
Free triiodothyronine (pmol/L)			
*Baseline*	3.5 ± 0.7	3.4 ± 0.7	0.5579
*Follow-up*	3.4 ± 0.6	3.6 ± 0.9	0.2849
*p-value (follow-up* vs. *baseline)*	0.5293	0.3101	-
FSH (U/L)			
*Baseline*	72 ± 24	69 ± 22	0.5928
*Follow-up*	70 ± 26	52 ± 20	**0.0021**
*p-value (follow-up* vs. *baseline)*	0.7428	**0.0014**	-
LH (U/L)			
*Baseline*	52 ± 16	53 ± 12	0.7715
*Follow-up*	54 ± 14	47 ± 13	**0.0364**
*p-value (follow-up* vs. *baseline)*	0.5852	0.0523	-
Estradiol (pg/mL)			
*Baseline*	17 ± 4	18 ± 4	0.3064
*Follow-up*	18 ± 5	19 ± 6	0.4540
*p-value (follow-up* vs. *baseline)*	0.3658	0.4216	-
Progesterone (ng/mL)			
*Baseline*	0.47 ± 0.20	0.52 ± 0.20	0.3064
*Follow-up*	0.49 ± 0.22	0.50 ± 0.24	0.8584
*p-value (follow-up* vs. *baseline)*	0.6162	0.7102	-
Anti-Müllerian hormone (pmol/L) ^a^			
*Baseline*	0.55 ± 0.65	0.50 ± 0.70	0.8305
*Follow-up*	0.60 ± 0.57	0.56 ± 0.68	0.8537
*p-value (follow-up* vs. *baseline)*	0.8130	0.8015	-
Prolactin (ng/mL)			
*Baseline*	14.9 ± 6.2	15.5 ± 5.0	0.5939
*Follow-up*	13.8 ± 6.0	14.2 ± 5.8	0.7807
*p-value (follow-up* vs. *baseline)*	0.3843	0.3258	-
ACTH (pg/mL)			
*Baseline*	35 ± 10	31 ± 16	0.2208
*Follow-up*	39 ± 15	36 ± 11	0.3504
*p-value (follow-up* vs. *baseline)*	0.2003	0.1380	-
Insulin-like growth factor-1 (ng/mL)			
*Baseline*	120 ± 30	112 ± 43	0.3769
*Follow-up*	125 ± 26	122 ± 46	0.7417
*p-value (follow-up* vs. *baseline)*	0.4653	0.3578	-
hsCRP (mg/L)			
*Baseline*	3.1 ± 0.8	2.0 ± 0.5	**<0.0001**
*Follow-up*	2.9 ± 0.8	1.0 ± 0.4	**<0.0001**
*p-value (follow-up* vs. *baseline)*	0.3064	**<0.0001**	-

Group A: postmenopausal women with autoimmune thyroiditis. Group B: postmenopausal women without thyroid pathology. The data are presented as the mean ± standard deviation. Statistically significant results are marked in bold. ^a^ Analysis of samples of 17 women from each group. Abbreviations: ACTH—adrenocorticotropic hormone; FSH—follicle-stimulating hormone; HOMA1-IR—the homeostatic model assessment 1 of insulin resistance ratio; hsCRP—high-sensitivity C-reactive protein; LH—luteinizing hormone; TgAb—thyroglobulin antibodies; TPOAb—thyroid peroxidase antibodies.

**Table 3:** Percentage changes from baseline in investigated variables.

Variable	Group A	Group B	*p*-Value (A vs. B)
Δ Glucose	−4 ± 4	−11 ± 5	**<0.0001**
Δ Insulin	−21 ± 19	−42 ± 20	**<0.0001**
Δ HOMA1-IR	−25 ± 20	−49 ± 25	**<0.0001**
Δ TPOAb	−11 ± 16	−7 ± 19	0.3512
Δ TgAb	−13 ± 20	−11 ± 25	0.7168
Δ Thyrotropin	−4 ± 10	−10 ± 20	0.1225
Δ Free thyroxine	3 ± 5	2 ± 8	0.5386
Δ Free triiodothyronine	−3 ± 14	5 ± 25	0.1283
Δ FSH	−3 ± 11	−25 ± 18	**0.0001**
Δ LH	4 ± 9	−11 ± 20	**0.0002**
Δ Estradiol	6 ± 10	6 ± 12	1.0000
Δ Progesterone	4 ± 18	−4 ± 23	0.1150
Δ Anti-Müllerian hormone ^a^	9 ± 14	12 ± 20	0.6158
Δ Prolactin	−7 ± 8	−8 ± 6	0.5618
Δ ACTH	11 ± 12	16 ± 19	0.1988
Δ Insulin-like growth factor-1	4 ± 11	9 ± 16	0.1380
Δ hsCRP	−6 ± 10	−50 ± 24	**<0.0001**

Group A: postmenopausal women with autoimmune thyroiditis. Group B: postmenopausal women without thyroid pathology. The data are presented as the mean ± standard deviation. Statistically significant results are marked in bold. ^a^ Analysis of samples of 17 women from each group. Abbreviations: ACTH—adrenocorticotropic hormone; FSH—follicle-stimulating hormone; HOMA1-IR—the homeostatic model assessment 1 of insulin resistance ratio; hsCRP—high-sensitivity C-reactive protein; LH—luteinizing hormone; TgAb—thyroglobulin antibodies; TPOAb—thyroid peroxidase antibodies.

**Table 4:** The impact of complying with the lifestyle modification program on glucose homeostasis markers, thyroid antibodies, hormones, and low-grade inflammation in euthyroid women with autoimmune thyroiditis and prediabetes.

Variable	Baseline	Follow-Up (Six Months Later)
Glucose (mmol/L)	6.11 ± 0.50	6.00 ± 0.61
Insulin (mU/L)	15.7 ± 5.5	15.2 ± 5.3
HOMA1-IR	4.2 ± 1.2	4.0 ± 1.4
TPOAb (IU/mL)	946 ± 345	910 ± 368
TgAb (IU/mL)	880 ± 355	868 ± 378
Thyrotropin (mIU/L)	2.5 ± 1.1	2.5 ± 1.0
Free thyroxine (pmol/L)	14.8 ± 2.5	15.3 ± 2.7
Free triiodothyronine (pmol/L)	3.4 ± 0.7	3.6 ± 0.8
FSH (U/L)	78 ± 24	82 ± 26
LH (U/L)	55 ± 18	57 ± 16
Estradiol (pg/mL)	16 ± 5	16 ± 6
Prolactin (ng/mL)	13.9 ± 5.1	14.3 ± 5.8
hsCRP (mg/L)	3.2 ± 0.9	3.1 ± 0.8

The data are presented as the mean ± standard deviation. Progesterone, anti-Müllerian hormone, ACTH, and insulin-like growth factor-1 were not determined in this population. Abbreviations: ACTH—adrenocorticotropic hormone; FSH—follicle-stimulating hormone; HOMA1-IR—the homeostatic model assessment 1 of insulin resistance ratio; hsCRP—high-sensitivity C-reactive protein; LH—luteinizing hormone; TgAb—thyroglobulin antibodies; TPOAb—thyroid peroxidase antibodies.

**Table 5:** The impact of complying with the lifestyle modification program on glucose homeostasis markers, thyroid antibodies, hormones, and low-grade inflammation in women with prediabetes and without thyroid pathology.

Variable	Baseline	Follow-Up (Six Months Later)
Glucose (mmol/L)	6.11 ± 0.65	6.00 ± 0.72
Insulin (mU/L)	14.8 ± 4.2	13.5 ± 4.0
HOMA1-IR	4.2 ± 1.5	3.6 ± 1.3
TPOAb (IU/mL)	14 ± 10	15 ± 12
TgAb (IU/mL)	19 ± 12	17 ± 11
Thyrotropin (mIU/L)	2.3 ± 0.9	2.2 ± 0.8
Free thyroxine (pmol/L)	14.2 ± 2.3	14.6 ± 2.9
Free triiodothyronine (pmol/L)	3.5 ± 0.8	3.5 ± 0.7
FSH (U/L)	76 ± 25	74 ± 26
LH (U/L)	57 ± 18	55 ± 16
Estradiol (pg/mL)	16 ± 6	17 ± 5
Prolactin (ng/mL)	14.7 ± 5.8	15.2 ± 6.0
hsCRP (mg/L)	1.9 ± 0.7	1.7 ± 0.8

The data are presented as the mean ± standard deviation. Progesterone, anti-Müllerian hormone, ACTH, and insulin-like growth factor-1 were not determined in this population. Abbreviations: ACTH—adrenocorticotropic hormone; FSH—follicle-stimulating hormone; HOMA1-IR—the homeostatic model assessment 1 of insulin resistance ratio; hsCRP—high-sensitivity C-reactive protein; LH—luteinizing hormone; TgAb—thyroglobulin antibodies; TPOAb—thyroid peroxidase antibodies.

## Data Availability

The data that support the findings of this study are available from the corresponding author upon reasonable request.

## Abbreviations

ACTH—adrenocorticotropic hormone; AMPK—adenosine 5′-monophosphate-activated protein kinase; FSH—follicle-stimulating hormone; HOMA1-IR—the homeostatic model assessment 1 of insulin resistance ratio; hsCRP—high-sensitivity C-reactive protein; LH—luteinizing hormone; TgAb—thyroglobulin antibodies; TPOAb—thyroid peroxidase antibodies.
